# Atrial fibrillation reduction by renal sympathetic denervation: 12 months’ results of the AFFORD study

**DOI:** 10.1007/s00392-018-1391-3

**Published:** 2018-11-10

**Authors:** Lida Feyz, Dominic A. Theuns, Rohit Bhagwandien, Mihai Strachinaru, Isabella Kardys, Nicolas M. Van Mieghem, Joost Daemen

**Affiliations:** Department of Cardiology, Thoraxcenter, Erasmus University Medical Center, Room Rg6, P.O. Box 2040, 3000 CA Rotterdam, The Netherlands

**Keywords:** Renal sympathetic denervation, RDN, Atrial fibrillation, AF, Implantable cardiac monitor, Quality of life

## Abstract

**Aim:**

The purpose of this pilot study was to assess whether renal sympathetic denervation (RDN) decreases atrial fibrillation (AF) burden in hypertensive patients with symptomatic AF at 6- and 12-month follow-up, as measured using an implantable cardiac monitor (ICM).

**Methods and results:**

A total of 20 patients with symptomatic paroxysmal or persistent AF (EHRA ≥ II) and primary hypertension with a mean office systolic blood pressure (BP) of > 140 mmHg were enrolled. After enrolment, an ICM was implanted 3 months pre-RDN to monitor AF burden. Quality of life (QOL) was assessed using the Atrial Fibrillation Effect on QualiTy-of-life (AFEQT) questionnaire. Mean age was 64 ± 7 years and 55% were females. AF burden in min/day decreased from a median (IQR) of 1.39 (0–11) pre-RDN to 0.67 (0–31.6) at 6 months (*p* = 0.64) and to 0.94 (0–6.0) at 12 months (pre-RDN vs. 12 months; *p* = 0.03). QOL improved significantly at both 6 months (+ 11 ± 15 points, *p* = 0.006) and 12 months (+ 10 ± 19, *p* = 0.04) as compared to pre-RDN. Office BP decreased significantly at 12-month follow-up (− 20 ± 19/− 7 ± 10 mmHg), p < 0.01) as compared to pre-RDN. Ambulatory BP decreased − 7 ± 16/− 3 ± 9 mmHg (*p* > 0.05) at 12-month follow-up as compared to pre-RDN.

**Conclusion:**

This pilot study suggests that RDN might be able to decrease AF burden in min/day as measured using an ICM, with a positive effect on QOL. Large-scale randomized trials are needed to prove the definite value of RDN in hypertensive patients with atrial fibrillation.

**Electronic supplementary material:**

The online version of this article (10.1007/s00392-018-1391-3) contains supplementary material, which is available to authorized users.

## Introduction

AF is the most common arrhythmia worldwide, occurring in 2–3% of the adult population [1]. The incidence of AF, along with an inherent risk for thromboembolic events, increases with age and the presence of hypertension [2]. Vice versa, hypertension is the most common cardiovascular condition responsible for the development and recurrence of AF [3]. AF is associated with an impaired quality of life (QOL) and is known to increase the risk of hospitalization by two- to threefold resulting in increased health care costs [4]. In general, the current treatment options for AF can be divided into either pharmacological and/or ablation therapy (i.e. pulmonary vein isolation, PVI), performed either by percutaneous or surgical techniques. Despite improving tools and techniques, many patients remain symptomatic and side effects of pharmacological treatment are common with high recurrence rates following catheter ablation, especially in patients with hypertension [5–7]. Furthermore, major complications as tamponade and stroke have been reported in up to 4.5% of the patients treated with percutaneous techniques, while the impact on survival associated with any of the therapies mentioned above is still disputed [8]. Previous pathophysiological studies described a correlation of AF burden to hyperactivity of the sympathetic nervous system (SNS) and demonstrated that, by modulating the SNS directly, AF control might improve significantly [9–11]. While renal sympathetic denervation (RDN) has been studied to help control hypertension [12], its potential value in improving signs and symptoms of AF is currently unknown. The aim of the present pilot study was to assess if RDN decreases occurrence and symptoms of AF in patients with symptomatic paroxysmal or persistent AF at 6- and 12-month follow-up.

## Methods

### Study design and patient population

This study is a single-arm pilot study including 20 patients. Patients were eligible for enrolment if all of the following inclusion criteria were met: paroxysmal or persistent AF, hypertension (mean office systolic BP ≥ 140 mmHg), use of ≥ 2 antihypertensive drugs, age ≥ 18 years, estimated glomerular filtration rate (eGFR) > 45 ml/min/1.73 m^2^. Paroxysmal AF was defined as an episode of AF that terminated spontaneously in less than 7 days. Persistent AF was defined as AF that fails to terminate in 7 days and an intervention was needed to restore sinus rhythm (pharmacologic or electrical cardioversion).

Patients with permanent AF, renal artery abnormalities, first episode of AF, comorbidities with a life expectancy of less than 1 year or unwillingness to undergo RDN or ICM implantation or follow-up visits were excluded. Secondary causes for hypertension were excluded prior to enrolment. The study was approved by our local ethics committee and all patients provided written informed consent (trialregister.nl, NTR number: NTR5329).

### Study measurements and endpoints

Clinical and laboratory data were obtained at 1, 3, 6 and 12 months post-RDN; annual follow-up will be continued up to 3 years. The primary efficacy objective, AF burden, was measured with the SJM Confirm DM2102 ICM (St. Jude Medical, St Paul, MN, USA) featuring both an automatic and a manual activation trigger, AF triggers, heart rate histograms, mean heart rate and ventricular rate response (VRR) monitoring during AF. The primary safety objective was defined as a composite of death from cardiovascular causes, stroke, major access site bleeding, acute kidney injury or renal artery stenosis.

Serious adverse events (SAE’s) and adverse events reported spontaneously by the subject or observed at each follow-up or any time in between, were recorded by the investigator. A SAE was defined as follows: any untoward medical occurrence, or effect, which in any dose results in events that were fatal or life-threatening, or that required a prolonged hospitalization; as well as any other important medical event that required intervention.

Secondary outcomes included change in office BP, ambulatory blood pressure measurements (ABPM), change in 24 h-holter monitoring, change in echocardiographic parameters (change in left ventricular (LV) and left atrial (LA) volumes and dimensions and change in LV diastolic function) and QOL measurements (using the AFEQT questionnaire). The AFEQT questionnaire is an AF-specific health related QOL questionnaire designed to be used in different clinical settings and for research purposes to assess the impact of AF on patients QOL. Overall or subscale scores range from 0 (complete disability or limitations) to 100 (no disability or limitations) [13].

Office BP was measured at each follow-up visit with an automatic blood pressure monitor (Omron M10-IT). ABPM was performed using the Ultralite Ambulatory Blood Pressure monitor (Spacelabs Healthcare, model 90217A) and 24 h-holter monitoring was performed with the evo digital recorder (Del Mar Reynolds, Spacelabs Healthcare) to assess changes in (supra) ventricular ectopic beats (S)VE beats.

We aimed to maintain the antihypertensive and antiarrhythmic drug regimen during the course of the study in all patients; however, changes were allowed in case of hypotension, hypertension or frequent AF episodes.

### ICM implantation and interrogation

ICM [SJM Confirm DM2102 (St. Jude Medical, St Paul, MN, USA)] implantation was performed by an electrophysiologist under local anesthesia 3 months pre RDN. A small incision (about 2–3 cm) was made lateral to the sternum at the level of the fourth and the fifth intercostal spaces. After the procedure, patients were instructed to use the activator in case of symptoms. To measure AF burden, the ICM was interrogated at each study visit per protocol by an expert (DT) to obtain the following parameters: functional status of the device, device battery level, analysis of any abnormal heart rhythms (AF, tachycardia, bradycardia and asystole) along with the highest VRR during AF episodes.

Final AF burden was assessed by confirming the AF episodes on the ECG readings to prevent false positive and false negative results. The minimum arrhythmia duration for an appropriate AF episode to be recorded was 30 s and a tachycardia cut-off rate of 120 bpm was applied. Final outcomes were based on 3-month intervals; pre-RDN (0–3 months pre- procedure), 6 months (3–6 months post-RDN) and 12 months (9–12 months post-RDN). AF burden was defined as the average minutes/day spent in AF. Prior to procedure a total of 2/20 patients with persistent AF progressed to permanent AF when using data derived from the long-term ECG recordings through ICM, both patients were excluded from the assessment of the primary outcome due to inequivalent burdens of AF as compared to patients with paroxysmal or persistent AF.

### RDN procedure

All patients were preloaded with 300 mg aspirin, if naïve, and advised to continue with aspirin for at least 1 month. Pre-procedurally, 100 IU heparin/kg was administered to achieve an active clotting time > 250 s. All procedures were performed under conscious sedation. After administration of local anesthesia, common femoral artery access was achieved by an ultrasound-guided puncture and a 6-Fr sheath was then introduced. Under fluoroscopic guidance, the short 6-Fr sheath was exchanged for an 8-Fr RDN or an IMA-tipped guiding sheath, to accommodate the St. Jude EnligHTN™ system. After smoothly engaging the renal arteries by using a no-touch technique with the help of a standard high-torque BHW coronary guidewire, selective renal artery angiograms were made and an appropriate basket size was chosen (small basket 4.0–5.5 mm diameter/large basket 5.5–8.0 mm diameter). The BHW guidewire was exchanged for the EnligHTN™ ablation catheter with its tip proximal to the bifurcation of the main renal artery. The basket catheter, containing four bipolar Platinum–Iridium electrodes, was then opened with the impedance of each electrode on the basket monitored. After a total of four ablations were performed successfully the basket was collapsed and retracted proximally while another four ablations were performed in the same artery, with the intention to achieve at least eight successful ablations per artery.

### Statistical analysis

Continuous variables were expressed as mean ± standard deviation (SD) when normally distributed; non-normally distributed variables were presented as median [interquartile range, IQR]. Categorical variables were expressed as percentages. Continuous variables were using Student's* t* test. Categorical variables were compared with the Chi square test or Fisher’s Exact test when appropriate. The Wilcoxon signed-rank test or McNemar’s test were performed to analyse the AF burden. Spearman correlation coefficient was used to evaluate the relationship between blood pressure drop and the change in AF burden. The Friedman test was performed to analyse EHRA class. All statistical tests are 2-tailed. A *p* value < 0.05 was considered statistically significant. Statistical analysis was performed using SPSS statistical analysis (version 21.0).

## Results

**Fig. 1:**
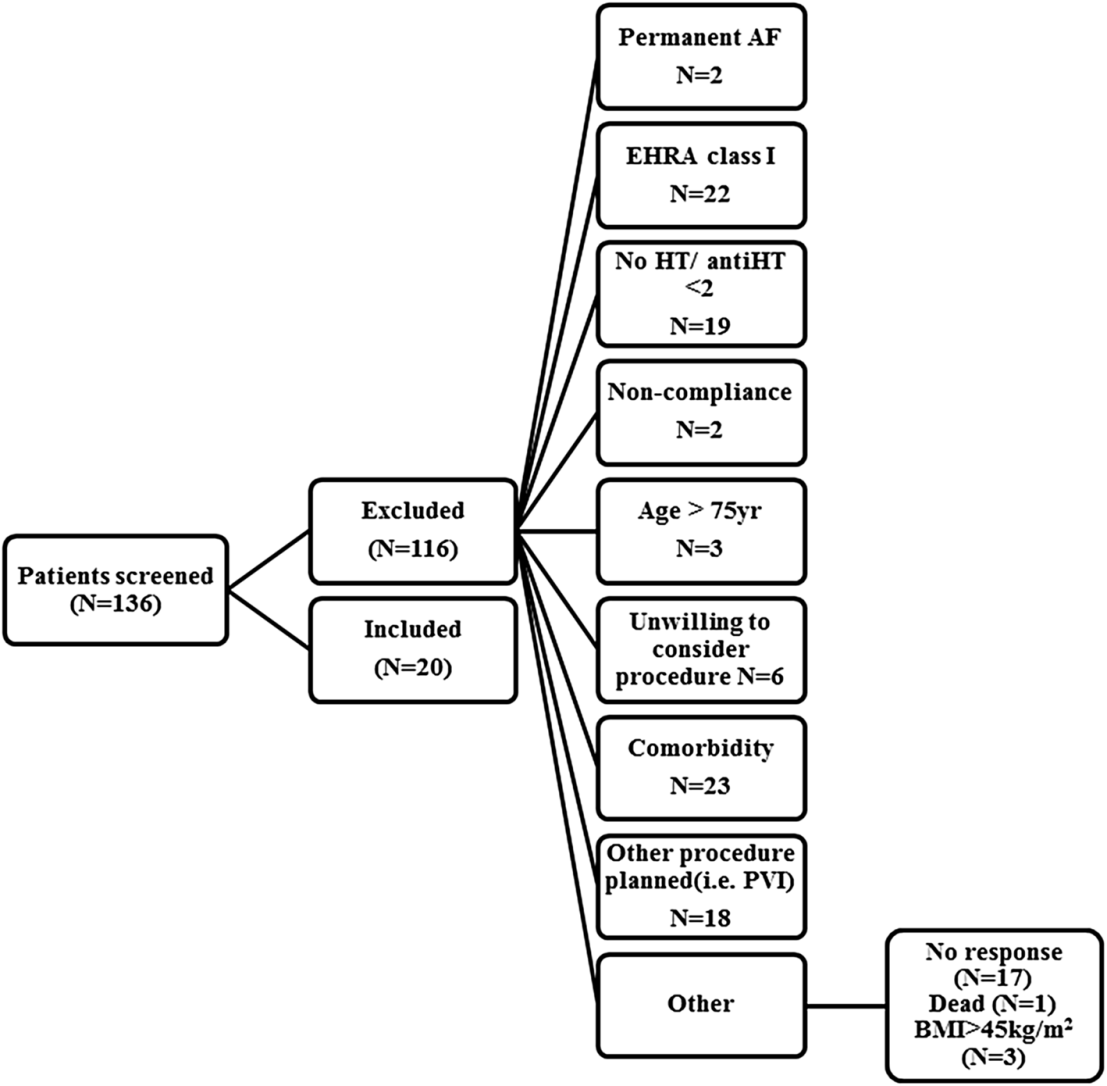
Screening process based on inclusion- and exclusion criteria. *AF* atrial fibrillation, *antiHT* antihypertensive medication, *BMI* body mass index, *EHRA* European Heart Rhythm Association, *HT* hypertension, *PVI* pulmonary vein isolation

Between July 2014 and February 2016 a total of 136 patients were screened for eligibility. Twenty patients (14.7%) met the inclusion criteria (Fig. 1). Baseline characteristics are presented in Table 1. In brief, nine patients were males and mean age was 64 ± 7 years. Mean office blood pressure was 153/88 mmHg. Most patients were on rhythm control (19/20) and 20% of the patients had a history of pulmonary vein isolation. Timing between the RDN- and the previous PVI-procedure in 4/20 patients was 916 ± 116 days. Based on ambulatory BP measurements a total of 11 patients had essential hypertension according the definitions of the European Society of Hypertension [14] and nine patients had white coat uncontrolled hypertension. None of the patients had a history of obstructive sleep apnea syndrome.

**Table 1:** Baseline characteristics of the study population

Total study population (*N* = 20)	
Age, years	64 ± 7
Male *n*, (%)	9 (45)
BMI (kg/m^2^)	30.7 ± 5.6
Paroxysmal AF *n*, (%)	18 (90)
EHRA class II	15 (75)
EHRA class III	5 (25)
Mean heart rate (bpm)	71 ± 15
Office BP (mmHg)	153 ± 17/88 ± 11
Ambulatory BP (mmHg)	130 ± 15/77 ± 9
Cardiovascular risk factors *n*, (%)	
Diabetes	2 (10)
Hypertension	20 (100)
Dyslipidemia	8 (40)
Smoking	1 (5)
Family history of IHD	4 (20)
Cardiovascular history *n*, (%)	
Prior PVI	4 (20)
Prior CVA	2 (10)
Antiarrhythmic drugs *n*, (%)	
Class I	4 (20)
Class II	1 (5)
Class III	12 (60)
Class V	2 (10)
Antihypertensive drugs *n*, (%)	
ACE-i	7 (35)
ARB	8 (40)
Beta-blockers^a^	18 (90)
CCB	10 (50)
Alfa-blocker	3 (15)
Diuretics	13 (65)

Values are mean ± SD or *n* (%)

*AF* atrial fibrillation, *BMI* body mass index, *BP* blood pressure, *CVA* cerebrovascular accident, *EHRA* European Heart Rhythm Association, *IHD* ischemic heart disease, *PVI* pulmonary vein isolation

^a^2/20 patients were intolerant for beta−blockers

AF burden decreased at 6 and 12 months. AF burden (min/day) was 1.39 (0–10.9) pre-RDN vs. 0.67 (0–31.6) at 6 months (*p* = 0.64) and 0.94 (0–6.0) at 12 months (*p* = 0.03). Changes in AF episodes and change in the total minutes in AF are presented in Table 2.

Two patients progressed from persistent AF to permanent AF prior to RDN and were excluded from AF burden analysis. Both patients underwent failed attempts to restore sinus rhythm by ECV. Despite persistent EHRA class II on rate control both patients declined PVI during the course of the study.A sub analysis in patients with a history of PVI and an additional RDN showed a numerical decrease in AF burden min/day at 6 months and 1 year follow-up, from a median of 973 min (11.1–1440) to 12.9 min (3.7–22.8) at 6 months to 1.57 (0.5–27.3) at 1 year (*p* > 0.05 for both).

**Table 2:** AF burden on ICM monitors pre-RDN vs. 6- and 12-month follow-up

	Pre-RDN	6 months	12 months	p*	p**
AF episodes (*n*)	1 (0–11)	1 (0–11)	3 (0–16)	0.84	0.31
Total episodes AF (min)	125 (2–978)	44 (0–2833)	84 (0–544)	0.64	0.03
AF min/day	1.39 (0–10.9)	0.67 (0–31.6)	0.94 (0–6.0)	0.64	0.03
Highest VRR (bpm)	127 (105–145)	117 (104–141)	106 (75–126)	0.09	0.01

Values are in median [IQR]

*AF* atrial fibrillation, *ICM* implantable cardiac monitor, *VRR* ventricular rate response during AF

*Between pre-RDN vs. 6 months, **pre-RDN vs. 12 months. Results are based on 18/20 patients, and patients with permanent AF were excluded

Renal function remained unchanged at both 6- and 12-month follow-up, eGFR (ml/min) pre-RDN was 83 ± 20 vs. 86 ± 21 at 6 months (*p* = 0.23) and 86 ± 23 at 12 months (*p* = 0.14). No cases of cardiovascular death, stroke, major access site bleeding, acute kidney injury or renal artery stenosis were reported. One peri-procedural complication was reported involving a renal artery dissection that resolved after balloon dilatation.

Office systolic BP decreased from 153 ± 17 mmHg pre-procedure to 148 ± 17 mmHg at 6 months (*p* = 0.13) and to 133 ± 16 at 12-month follow-up (*p* < 0.01) (Table 3). No correlation was found between Δ BP (ABPM) and Δ AF burden (in terms of min/day or episodes); at 12 months (Δ number of AF episodes and Δ mean 24 h systolic ABPM *r* = − 0.09; *p* = 0.74 and ΔAF min/day and Δ mean 24 h systolic ABPM, *r* = 0.30; *p* = 0.28).

**Table 3:** Office and ambulatory blood pressure change pre-RDN vs. 6- and 12-month follow-up

	Pre-RDN	6 months	12 months	p*	p**
Office systolic BP (mmHg)	153 ± 17	148 ± 17	133 ± 16	0.13	< 0.01
Office diastolic BP (mmHg)	89 ± 10	81 ± 11	81 ± 10	0.006	0.007
24 h ABPM systolic (mmHg)	131 ± 16	121 ± 9	124 ± 11	0.007	0.07
24 h ABPM diastolic (mmHg)	78 ± 9	72 ± 6	74 ± 9	0.006	0.16

Values are mean ± SD

*ABPM* ambulatory blood pressure measurement, *BP* blood pressure

*Pre−RDN vs. 6 months

**Pre−RDN vs. 12 months

**Fig. 2:**
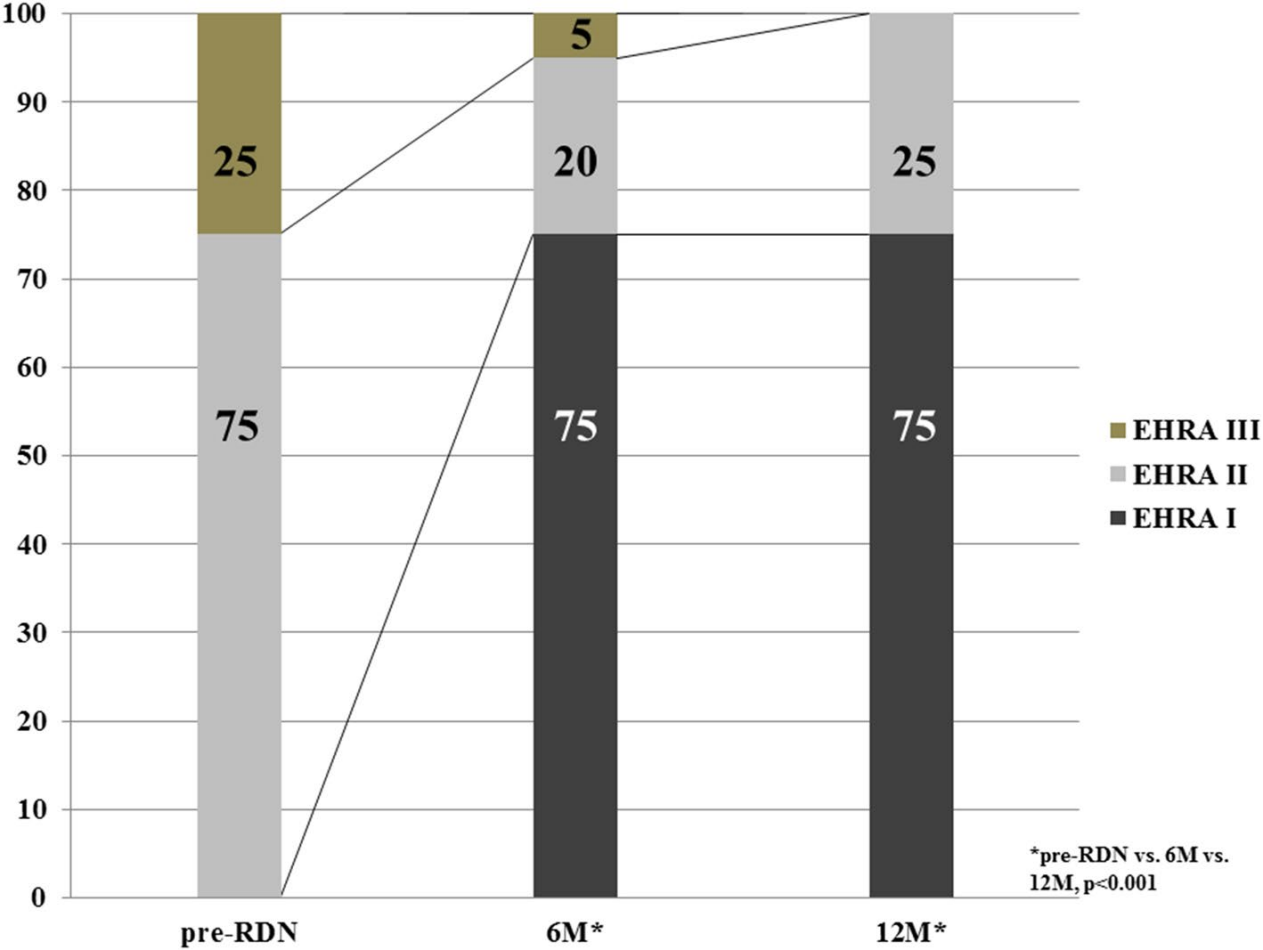
Change in EHRA class at follow-up visits (6- and 12 months results). *EHRA* European Heart Rhythm Association, *pre-RDN* prior to renal sympathetic denervation, *6M* 6 months, *12M* 12 months

Mean heart rate on 24 h-holter monitoring remained unchanged at 6- months (− 5 ± 14 bpm; *p* = 0.15) and 12-month follow-up (− 1 ± 14 bpm; *p* = 0.63) as compared to baseline. A numerical decrease was seen in SVE beats at 6- and 12-month follow-up as compared to pre-procedure. VE beats remained unchanged during follow-up (Table 4). EHRA class improved significantly at both 6 and 12 months as compared to pre-RDN (*p* < 0.01) (Fig. 2).

**Table 4:** 24 h-holter monitoring for (S)VE beats

	Pre-RDN	6 months	12 months	p*	p**
Heart rate (bpm)	71 ± 15	66 ± 8	70 ± 12	0.15	0.63
SVE (beats)	187 (82–948)	137 (43–1096)	79 (13–763)	0.36	0.05
VE (beats)	35 (3–153)	22 (3–86)	42 (5–134)	0.57	0.73

Values are mean ± SD or are median [IQR]

*(S)VE* (supraventricular)ectopic beats

*Pre−RDN vs. 6 months

**Pre−RDN vs. 12 months

None of the patients underwent PVI within 1 year follow-up post-RDN.

**Fig. 3:**
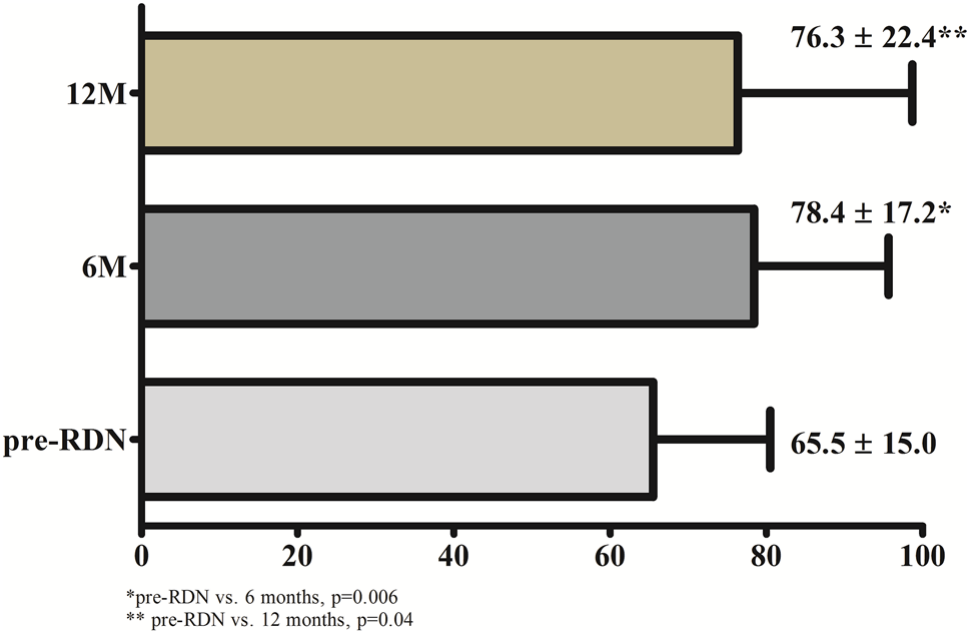
AFEQT questionnaire. *pre-RDN* prior to renal sympathetic denervation, *6M* 6 months, *12M* 12 months

No change was found in LV and LA volumes and dimensions at 6- and 12 months post RDN (Supplement, Table S1). QOL improved significantly at both follow-up visits (as compared to pre-procedure; +11 ± 15 points at 6 months, *p* < 0.01 and + 10 ± 19 at 12 months, *p* = 0.04) (Fig. 3).

A total of 3/20 (15%) patients underwent an ECV post RDN due to recurrence of AF at 6 months; the average period to perform the first ECV post-RDN in these patients was 134 ± 83 days. In two out of these three patients a second ECV was done and one patient received a total of three ECV’s during follow-up.

Despite efforts in maintaining initial drug regimens during the course of the study, antiarrhythmic drugs or drug dosages were increased in 3/20 patients (these were different patients from who underwent an ECV) and decreased in 6/20 patients. Antihypertensive medication was increased in 2/20 patients and decreased or stopped in 4/20 patients.

## Discussion

This single-center pilot study suggests that RDN was able to significantly decrease AF burden in min/day as measured using an ICM, together with an improvement in QOL. Finally, RDN appeared safe with a positive effect on blood pressure.

Sympathetic hyperactivity has been considered an important source for the induction and maintenance of arrhythmias [15]. Previous studies provided evidence for the presence of increased atrial sympathetic activity in both persistent and paroxysmal AF, suggesting autonomic remodeling may be part of the atrial substrate for AF [16, 17].

Pathophysiological studies demonstrated that by modulating the SNS through RDN, AF control might improve [18]. Linz and colleagues demonstrated the effect of RDN on heart rate and VRR in pigs with permanent AF; the authors described a reduction of 24% in VRR in the treated pigs vs. sham [19]. Furthermore, the potential effects of RDN on ventricular electrophysiological properties should be mentioned. Huang and colleagues demonstrated that RDN could alter the ventricular effective refractory period (ERP) and action potential duration (APD) in eight dogs as compared to a sham operation group (*N* = 8) [20]. The authors described a prolongation of ventricular APD after RDN, which hypothetically could prevent the occurrence of fatal ventricular arrhythmias (VA). A multicenter registry reported that RDN appeared to be safe and efficacious in reducing VA burden in patients with chronic heart failure; the procedure was performed in patients with a few or no further therapeutic options [21]. Finally, successful anecdotal experience was achieved with RDN in patients with refractory vasospastic-induced ventricular tachycardia [22]. The findings, however, do suggest that RDN might be a potentially more appealing option to modify cardiac electrophysiological properties as compared to pivotal work on cardiac catheter ablation which proved to shorten the ERP and increase the incidence of premature ventricular complexes [23]. Of note, in the present study, no changes were observed over time in the incidence of ventricular ectopic beats.

Several recent randomized sham controlled trials proved the blood pressure lowering effect of RDN in both hypertensive patients taken off medication as well as in patients with uncontrolled hypertension, respectively [12, 24–26]. The multi-electrode EnligHTN™ catheter showed to decrease BP based on ABPM and to reduce renal norepinephrine in previous animal studies [27, 28]. Specifically related to the present human study, similar BP lowering effect was observed with the same catheter in patients with treatment-resistant hypertension [29].

Besides the antihypertensive effects of RDN, an experimental study by Tsai and colleagues showed the antiarrhythmic effects of RDN in six ambulatory dogs by measuring lower nerve activity at the level of the stellate ganglion with also a decrease in paroxysmal atrial tachycardia episodes and duration after RDN as compared to controls [10]. Moreover, two clinical studies assessed the antiarrhythmic effect of RDN in addition to PVI in hypertensive patients with symptomatic AF. One of these studies demonstrated a positive correlation between the decrease in mean BP and the decline in AF burden. A reduction of 5–10 mmHg in mean BP led to a 7% decrease in mean AF burden as measured with an ICM [30]. Unfortunately, in the present study, we were not able to show a clear correlation between the change in blood pressure and change in AF burden following RDN. Pokushalov and colleagues showed that RDN on top of PVI in patients with symptomatic AF and resistant hypertension reduced the incidence of AF recurrence rates significantly. At 1 year, based on 24 h-holter monitoring, 69% of the patients in the PVI + RDN were free of AF episodes, while in the PVI only group, only 29% of the patients remained free of AF episodes [6]. A dedicated prospective randomized controlled trial is currently ongoing to determine the efficacy of RDN on top of PVI in patients with hypertension [31] (Clinicaltrials.gov, NCT02115100).

It can be hypothesized that the antiarrhythmic effects of RDN could be due to a synergistic effect of (a) better BP control (partly) withdrawing an important risk factor for AF recurrence and (b) improving AF control by modifying electrophysiological settings like prolongation of the atrial effective refractory period [3, 32]. AF burden (in min/day and episodes) decreased significantly at 1 year follow-up; however, it did not reach statistical significance at 6 months’ follow-up. Although the latter could be due to the fact that the variability in AF burden at 6 months was high, an increasing effect of RDN over time could not be excluded. In the recently published SPYRAL-ON MED trial, BP reduction was greater at 6 months as compared with 3 months [33].

To the best of our knowledge, the present work is the first clinical study to demonstrate that RDN, without concomitant PVI, may reduce AF burden as measured using an ICM. Measuring AF burden with an ICM is superior to intermittent AF event monitoring using 24 h-holters or event recorders [34]. Monitoring treatment effect based on symptoms alone is unreliable since approximately 50% of AF recurrences proved to occur in asymptomatic patients [35]. Vice versa, Padeletti and colleagues showed a high intra-patient burden variability and demonstrated that only 52% of patient symptoms appear to be correlated to documented AF [36]. The latter was confirmed in our study in which we found a clear discrepancy between pre-procedural EHRA class and the actual AF episodes as measured using either 24 h-holter or ICM. Nevertheless, despite a large variability in AF burden between patients we were able to demonstrate a significant reduction in AF burden post RDN.

Of note, despite their known limitations, the vast majority of previous studies assessing the efficacy of either PVI or surgical ablation used intermittent and symptom-based monitoring (i.e. 24 h holter monitoring or event monitors) to demonstrate their effect [37].

Finally, we were not able to demonstrate any changes in echocardiographic parameters and diastolic function in our study population, which could be due to either the small sample size or advanced stages of LA dilatation prior to study participation.

## Limitations

Despite the positive results in this present study, there are several limitations that should be taken into account. First, it concerns a small study cohort (*N* = 20) in whom two patients progressed to permanent AF and were excluded from the analysis of AF burden. Second, in approximately 50% of the treated patients, we stopped or decreased drug dosages of antihypertensive and/or anti-arrhythmic drugs. Third, adherence to antihypertensive drugs was not measured and only confirmed by the physician at the outpatient clinic visit. Fourth, the ICM used in this study was not able to transmit wirelessly the ECG to the office, which could have led to an underestimation of the AF events in case of data overload and a lack of memory (which occurred in 1/20 patient). Fifth, RDN was performed with the EnligHTN™ ablation catheter which enabled the operator to ablate only in the main renal arteries and is currently no longer available for clinical use. Whether the use of current generation devices using different technologies as ultrasound, ethanol or RF sidebranch ablation would have resulted in more potent results remains to be determined. Finally, since no sham control group was included in the present pilot study, the potential of a placebo effect cannot be ruled out. Nevertheless, we showed the potential value of RDN in patients with paroxysmal AF and warrants the conduction of larger and sham controlled studies assessing the anti-arrhythmic effect of RDN.

## Conclusion

This pilot study suggests that RDN was safe and able to decrease AF burden in min/day as measured using an ICM at 12-month follow-up, together with an improvement in QOL in patients with symptomatic paroxysmal or persistent AF. Large-scale randomized trials are needed to demonstrate the value of RDN in hypertensive patients with AF.

## Electronic supplementary material

Below is the link to the electronic supplementary material.

Supplementary material 1 (DOCX 17 KB)
