# Supplementary material for: Atrial fibrillation reduction by renal sympathetic denervation: 12 months’ results of the AFFORD study
Source: Clin Res Cardiol. 2018 Nov 10;108(6):634–42. doi: 10.1007/s00392-018-1391-3 (PMC6529371; doi:10.1007/s00392-018-1391-3)
Supplement: Supplementary file 1 — Supplementary material 1 (DOCX 17 KB) [file 392_2018_1391_MOESM1_ESM.docx]

**Supplement**

**Table S1. Change in left ventricular and atrial volumes and diastolic function**

|  | pre-RDN | 6 months | 12 months | p* | p** |
| --- | --- | --- | --- | --- | --- |
| LA-dimension, mm | 44 ± 5 | 43 ± 5 | 44 ± 4 | 0.31 | 0.89 |
| LAVI, ml/m^2^ | 38 ± 8 | 39 ± 9 | 43 ± 12 | 0.58 | 0.17 |
| E, cm/s | 68 ± 18 | 73 ± 17 | 70 ± 25 | 0.02 | 0.53 |
| A, cm/s | 62 ± 20 | 65 ± 16 | 61 ± 18 | 0.38 | 0.56 |
| Septal E’, cm/s | 6.2 ± 1.3 | 6.4 ± 1.3 | 6.6 ± 1.3 | 0.65 | 0.24 |
| E/A ratio | 1.13 ± 0.4 | 1.09 ± 0.4 | 1.07 ± 0.4 | 0.57 | 0.49 |
| E/E’ ratio | 11.6 ± 3.4 | 11.9 ± 2.8 | 11.4 ± 4.7 | 0.58 | 0.91 |
| DET, ms | 220 ± 56 | 207 ± 39 | 219 ± 72 | 0.27 | 0.92 |
| LVEDD, mm | 54 ± 6 | 53 ± 5 | 52 ± 5 | 0.24 | 0.16 |
| LVESD, mm | 36 ± 5 | 32 ± 6 | 34 ± 7 | 0.04 | 0.56 |
| LVEF, % | 63 ± 7 | 63 ± 5 | 64 ± 6 | 0.96 | 0.53 |

^Values are mean ± SD . LA=left atrial, LAVI=left atrial volume indexed, E=peak early phase filling velocity, A=peak atrial phase filling velocity, E’=peak early wave velocity, DET=deceleration time, LVEDD=left ventricular end-diastolic dimension, LVESD=left ventricular end-systolic dimension, LVEF=left ventricular ejection fraction. *pre-RDN vs. 6 months . **pre-RDN vs. 12 months.^
